# Exploring potential therapeutic agents for lipopolysaccharide-induced septic cardiomyopathy based on transcriptomics using bioinformatics

**DOI:** 10.1038/s41598-023-47699-0

**Published:** 2023-11-23

**Authors:** Shaodan Feng, Kexin Cai, Siming Lin, Xiaojun Chen, Yuqing Luo, Jing Wang, Guili Lian, Zhihong Lin, Liangdi Xie

**Affiliations:** 1 Department of Emergency, The First Affiliated Hospital of Fujian Medical University, Fujian, Fuzhou, 350005 China; 2 Fujian Hypertension Research Institute, The First Affiliated Hospital of Fujian Medical University, Fujian, Fuzhou, 350005 China; 3 Clinical Research Center for Geriatric Hypertension Disease of Fujian Province, The First Affiliated Hospital of Fujian Medical University, Fujian, Fuzhou, 350005 China; 4 Department of Geriatrics, The First Affiliated Hospital of Fujian Medical University, Fujian, Fuzhou, 350005 China; 5 Branch of National Clinical Research Center for Aging and Medicine, The First Affiliated Hospital of Fujian Medical University, Fujian, Fuzhou, 350005 China; 6 Department of Geriatrics, National Regional Medical Center, Binhai Campus of the First Affiliated Hospital, Fujian Medical University, Fujian, Fuzhou, 350212 China

**Keywords:** Computational biology and bioinformatics, Immunology, Biomarkers, Cardiology, Pathogenesis

## Abstract

Septic cardiomyopathy (SCM) is a common and severe complication of sepsis, characterized by left ventricular dilation and reduced ejection fraction leading to heart failure. The pathogenesis of SCM remains unclear. Understanding the SCM pathogenesis is essential in the search for effective therapeutic agents for SCM. This study was to investigate the pathophysiology of SCM and explore new therapeutic drugs by bioinformatics. An SCM rat model was established by injection of 10 mg/kg lipopolysaccharide (LPS) for 24 h, and the myocardial tissues were collected for RNA sequencing. The differentially expressed genes (DEGs) between LPS rats and control (Ctrl) with the thresholds of |log2^fold change^|≥ 1 and *P* < 0.05. A protein–protein interaction (PPI) network was constructed based on the DEGs. The hub genes were identified using five algorithms of Cytoscape in the PPI networks and validated in the GSE185754 dataset and by RT-qPCR. The hub genes were analyzed by Gene ontology (GO) analysis and Kyoto Encyclopedia of Genes and Genomes (KEGG), as well as Gene set enrichment analyses (GSEA). In addition, the miRNAs of hub genes were predicted through miRWalk, and the candidate therapeutic drugs were identified using the Connectivity Map (CMAP) database. This study revealed the identified hub genes (*Itgb1, Il1b, Rac2, Vegfa*) and key miRNAs (rno-miR-541-5p, rno-miR-487b-3p, rno-miR-1224, rno-miR-378a-5p, rno-miR-6334, and rno-miR-466b-5p), which were potential biological targets and biomarkers of SCM. Anomalies in cytokine-cytokine receptor interactions, complement and coagulation cascades, chemokine signaling pathways, and MAPK signaling pathways also played vital roles in SCM pathogenesis. Two high-confidence candidate compounds (KU-0063794 and dasatinib) were identified from the CMAP database as new therapeutic drugs for SCM. In summary, these four identified hub genes and enrichment pathways may hold promise for diagnosing and treating SCM.

## Introduction

Sepsis is a life-threatening disease that results in organ dysfunction due to dysregulation of the host response^1^. Approximately 50% of patients with sepsis with some degree of myocardial damage are diagnosed with septic cardiomyopathy (SCM)^2^. SCM is a complication of sepsis and has a 70–90% mortality rate, posing a severe threat to the lives of patients^3^. There aren’t effective treatments for SCM. Active exploration of SCM pathogenesis and effective treatment options are vital parts of medical research.

It is believed that the occurrence and development of SCM are mainly the results of interactions between the immune and cardiovascular systems^4^. Upon a pathogen invading the body, the pattern of recognition receptors determines its invasive mechanism^5^. For example, toll-like receptors (TLRs) are expressed in both immune cells and other cells, such as cardiomyocytes, and interact with different molecular patterns associated with pathogens, leading to the activation of the nuclear factor-κB (NF-κB) and p38/MAPK signaling pathways, triggering a response of the inflammatory cascade and the production of large quantities of immunologic factors like interleukin 6 (IL-6), IL-8 and tumor necrosis factor (TNF)-α^6^. Furthermore, bacterial toxins, inflammatory factors, complement activation, mitochondrial damage, oxidative stress, and cardiomyocyte apoptosis can lead to systolic myocardial damage and impaired cardiomyocyte metabolism^7^.

SCM is a reversible cardiac dysfunction that spontaneously resolves when the primary disease is controlled. There is a lack of reliable clinical diagnostic indicators for patients with SCM, and few effective treatment methods. Moreover, patients with sepsis are heterogeneous, and conventional markers of myocardial injury are susceptible to other complications. In this study, a Connectivity Map (CMAP) was used to identify drugs with high therapeutic potential. Current drugs offer additional possibilities for SCM treatment.

Transcriptome sequencing (RNA-seq) is a rapid, accurate, and high-throughput method for analyzing entire transcripts in cells or tissues. RNA-seq technology has many applications, such as gaining insight into gene expression patterns in various biological processes identifying differentially expressed genes (DEGs), and exploring gene regulation mechanisms. In addition, this technology has received attention in many human diseases research fields, such as tumors, neurological diseases, and cardiovascular diseases, intending to reveal the mechanisms of disease onset and development at the transcriptome level^8–10^. RNA-seq technology can be used to explore the molecular mechanisms of SCM, including studying DEGs and non-coding RNAs, to identify new therapeutic targets.

In this study, RNA-seq was performed on the myocardial tissue of an SCM rat model injected with lipopolysaccharide (LPS) to obtain gene expression profiles. Through bioinformatics analysis of 633 DEGs (456 upregulated and 177 downregulated), four hub genes (*Il1b*, *Rac2*, *Vegfa*, *Itgb1*) were obtained. Four hub genes may help to screen biomarkers for early diagnosis, further understand the biomolecular mechanism of SCM, and provide new ideas for early diagnosis. Two high-confidence candidate compounds (KU-0063794 and dasatinib) were identified from the CMAP database as new therapeutic drugs for SCM.

## Materials and methods

### Animals

The study was approved by the Laboratory Animal Welfare and Ethics Committee of Fujian Medical University (approval number: MRCTA, ECFAHB of FMU [2022] 244, Fuzhou, China). All procedures were carried out following the ethical standards. All methods were reported following ARRIVE guidelines. All surgeries were performed with sodium pentobarbital anesthesia, and all efforts were made to minimize suffering. Ten male Sprague–Dawley rats weighing approximately 200 g were purchased from Beijing Huifang Biotechnology Co., Ltd. (Beijing, China) under the license SCXK (Beijing) 2019–0008. All rats were raised in the animal room with constant temperature (22 ± 2) °C, constant humidity (55 ± 5) %, and light for 12 h/day and were fed and watered ad libitum. After one week of adaptation, ten rats were randomly divided into the LPS group (n = 5), injected intraperitoneally with LPS (10 mg/kg), and the control (Ctrl) group (n = 5), injected with the same volume of 0.9% saline. LPS derived from Escherichia coli (055:B5) was acquired from Sigma (China). At the end of the experiment, the rats were sacrificed, and their blood and hearts were harvested for subsequent analysis.

### Echocardiography

Twenty-four hours after the LPS injection, 30 mg/kg pentobarbital sodium was injected intraperitoneally for anesthesia. Then, echocardiography was performed using a Vivid 7 Pro (GE, USA) diagnostic ultrasound scanner in the M-ultrasound mode. The left ventricular ejection fraction (LVEF) and left ventricular fractional shortening (LVFS) were recorded to assess SCM.

### Histologic examination

SCM was assessed based on heart histology and morphology. After 24 h of LPS stimulation, the rats were euthanized with pentobarbital, and the heart of each rat was collected immediately after blood collection. Left ventricular myocardial tissue was collected along the coronal plane of the largest transverse diameter of the left ventricle and placed in a 4% formaldehyde solution for 12 h. The tissue was fixed, dehydrated, paraffin-embedded, and thick-sectioned at 4 µm. The slides were incubated with hematoxylin for nuclear staining, treated with 0.5% hydrochloric acid alcohol, and stained with 1% eosin for approximately 3 min at room temperature. HE staining was used to observe the pathological changes in the myocardium under a light microscope (Nikon, Tokyo, Japan).

### Enzyme-linked immunosorbent assay (ELISA)

The concentrations of cardiac troponin I (c-TnI) and brain natriuretic peptide (BNP) in rat serum were measured using ELISA kits (Cloud-Clone Corp., Wuhan, China), and each procedure was performed according to the manufacturer's protocol.

### RNA extraction, purification, and sequencing

TRIzol reagent (Invitrogen, China) was used to extract total RNA from the myocardial tissue, and a Nanodrop^TM^ OneC spectrophotometer (Thermo Fisher Scientific Inc.) was used to determine RNA concentration and purity. RNA integrity was examined by 1.5% agarose gel electrophoresis. A wide range of RNA was measured and assayed using the Qubit^TM^ RNA Wide Range Assay Kit (Life Technologies, Q10210) and Qubit 3.0. High-quality total RNA was used to construct cDNA libraries, and RNA-seq was performed by Wuhan SeqHealth (Wuhan, China). After high-throughput sequencing of multiplexes using Illumina Novaseq6000, the raw sequences were processed as follows: (a) low-quality readings filtered by Trimomatic (version 0.36); (b) quality assurance with FastQC software; (c) mapping clean reads to the rat reference genome with STAR (version 2.5.3a); (d) calculation of transcript abundance using reads per kilobase per million Reads (RPKM); and (e) identification of DEGs using edgeR (version 3.12.1).

### Bioinformatic analysis

#### DEGs analysis

**Figure 1:**
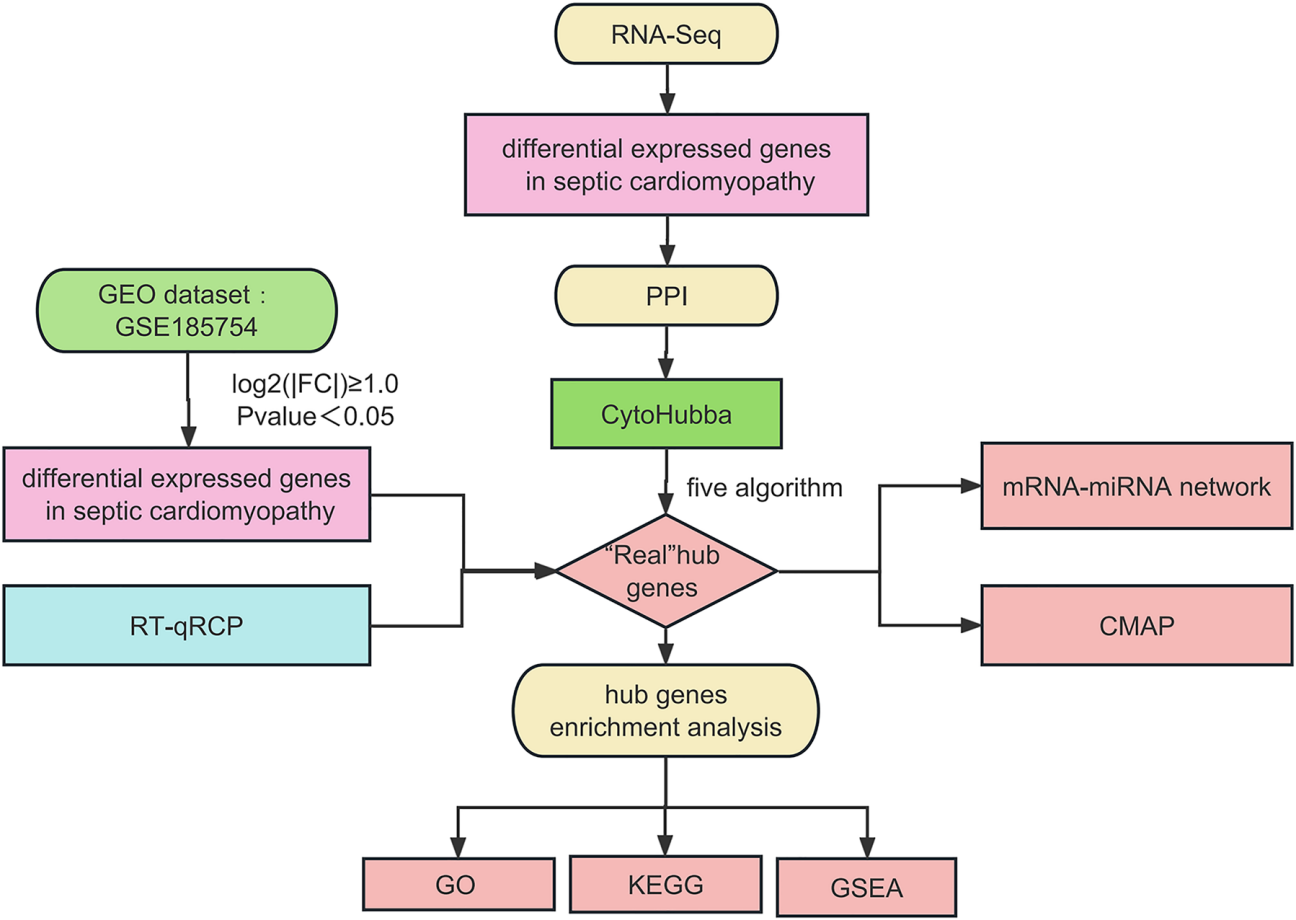
The flow chart of bioinformatics. *GEO* gene expression omnibus, *GSEA* gene set enrichment analysis, *GO* gene ontology, *KEGG* kyoto encyclopedia of genes and genomesm, *PPI* protein–protein interaction, *CMAP* connectivity map, *RT-qPCR* real-time quantitative polymerase chain reaction.

The edgeR package (version 3.12.1) was used to screen DEGs in different groups of the RNA-seq dataset, with the defined criteria of |log_2_^fold change^|≥ 1 and *P* < 0.05. Then, the "ggplot2" package of R software (version 3.3.6) was applied to draw a volcano map and box line diagram to show the expression patterns of DEGs. In addition, the "Complex Heat Map" of R software (version 2.2.0) was used to create a heat map to cluster with RPKM values and use red and blue colors to indicate upregulated and downregulated expressed genes respectively. To further investigate the DEGs between the LPS and Ctrl groups, bidirectional hierarchical clustering of genes and samples was performed, and the results were displayed using heat maps. The flow chart of the study was shown in Fig. 1.

#### Protein–protein interaction (PPI) network and hub gene identification

The PPI networks of DEGs were created using the STRING website (http://string.embl.de/). CytoHubba, a plug-in of Cytoscape (version 3.10.0), was carried out to filter the top 10 hub genes of DEGs according to five algorithms (MNC, degree, bottleneck, betweenness, and stress). Then, hub genes were visualized by the UpSetR package.

#### Database selection to validate hub genes

The GSE185754 dataset of the mouse mRNA expression profile was acquired from the Gene Expression Omnibus (GEO) database (https://www.ncbi.nlm.nih.gov/geo/) and included five LPS (intraperitoneal injection of 10 mg/kg for 24 h) and five Ctrl myocardium samples. "ggplot2" (version 3.3.6) of R software (version 4.2.2) was used to draw a boxed plot of hub genes to analyze further the differences between the RNA-seq and GSE185754 datasets.

#### Gene ontology (GO) and kyoto encyclopedia of genes and genomes (KEGG) pathway enrichment of DEGs

To comprehend the biological mechanisms of hub genes, GO and KEGG enrichment analyses were performed using "clusterProfiler"(version 3.14.3) and "ggplot2" (version 3.3.6). GO analysis included molecular function (MF), cellular components (CC), and biological processes (BP).

#### Gene set enrichment analysis (GSEA) of hub genes

The objectives of the GSEA were to evaluate RNA-seq dataset at the gene set levels, which were chosen based on a normalized enrichment score (NES) > 1.5 as well as *P* < 0.05. GSEA was mapped with the "clusterProfiler" and "msigdbr" packages of R software (version 4.2.1), relying on the log2 FC of hub genes.

#### Receiver operating characteristic curve (ROC) of hub genes

Receiver operating characteristic curve (ROC) was the core index of performance differentiation evaluation of medical diagnostic tests and predictive model.

#### Construction of mRNA-miRNA regulatory network

Hub genes were analyzed using the miRWalk database (http://mirwalk.umm.uni-heidelberg.de/) to predict target miRNAs. Seven algorithms of Cytoscape (DMNC, MNC, closeness, radiality, bottleneck, eccentricity, and clustering coefficient) were used to obtain the key miRNAs. Then, the key miRNAs were visualized by the UpSetR package.

#### Potential therapeutic drug studies in the connectivity map (CMAP)

CMAP (https://clue.io/) linked small molecules to gene expression profiles, and the degree of correlation between small molecules and genes was scored to identify a solid functional link between drugs and genes. Positive scores indicated a strong correlation between DEGs and reference genes. In contrast, negative scores suggested that the expression patterns of the upregulated and downregulated genes may differ from those of the reference genes. The 3D structures of small drug molecules were obtained from PubChem (https://pubchem.ncbi.nlm.nih.gov/).

### Cell culture

H9C2 cardiomyocytes were purchased from the American Type Culture Collection (ATCC, Manassas, VA, USA) and cultured in DMEM medium containing 10% fetal bovine serum at 37°C and 5% CO_2_ in a humidified incubator. When the cell density reached 70–80%, the cell was cultured at 1:3 ratio after digestion with 0.25% trypsin. The in vitro cell model of SCM was induced by treating H9C2 cells with 1 μg/ml LPS for 24 h^11^.

### Real-time quantitative polymerase chain reaction (RT-qPCR) of hub genes

RNA was extracted from myocardial tissues and cardiomyocytes of the LPS and Ctrl groups using TRIzol reagent (Vazyme Biotechnology, China) according to the manufacturer's protocol. After extraction, RNA from all the samples was reverse transcribed into cDNA by the Hifair®II 1st Strand cDNA Synthesis kit (YEASEN, China). The qPCR SYBR® Green Master Mix (No Rox) (YEASEN, China) was used for fluorescence quantification. All reactions were run in triplicate and performed on a Light Cycler 96 system (Roche, Switzerland). The housekeeping gene GAPDH served as a control. Relative levels of gene expression were calculated according to the 2^−△△Ct^ method. All primer sequences were shown in Table 1.

**Table 1:** Primer sequences in this study.

Primer	Sequence	Product size[bp]
IL1b-F	5′GCTACCTATGTCTTGCCCGT 3′	123
IL1b-R	5′TCACACACTAGCAGGTCGTC 3′	
Itgb1-F	5′ACAGATGAAGTGAACAGTGAAGAC 3′	167
Itgb1-R	5′GGACCTATCGCAGTTGAAGTTATC 3′	
VEGFA-F	5′TTACTGCTGTACCTCCACCAT 3′	139
VEGFA-R	5′CAGGACGGCTTGAAGATATACTC 3′	
Rac2-F	5′AGCTGGACCTTCGAGATGAC 3′	179
Rac2-R	5′TGCTCGGATTGCCTCATCG 3′	
GAPDH-F	5′ACGGCAAGTTCAACGGCACAG 3′	149
GAPDH-R	5′GAAGACGCCAGTAGACTCCACGAC 3′	

### Statistical analytics

Experiments were performed using five rats in each group. All in vitro experiments were repeated thrice. Data were presented as mean ± standard deviation (SD) and were analyzed using SPSS software (version 18.0). Student's t-test was used to compare the differences between the LPS and Ctrl groups. *P* < 0.05 was considered statistically significant.

## Results

### LPS caused changes in systemic reactions

**Figure 2:**
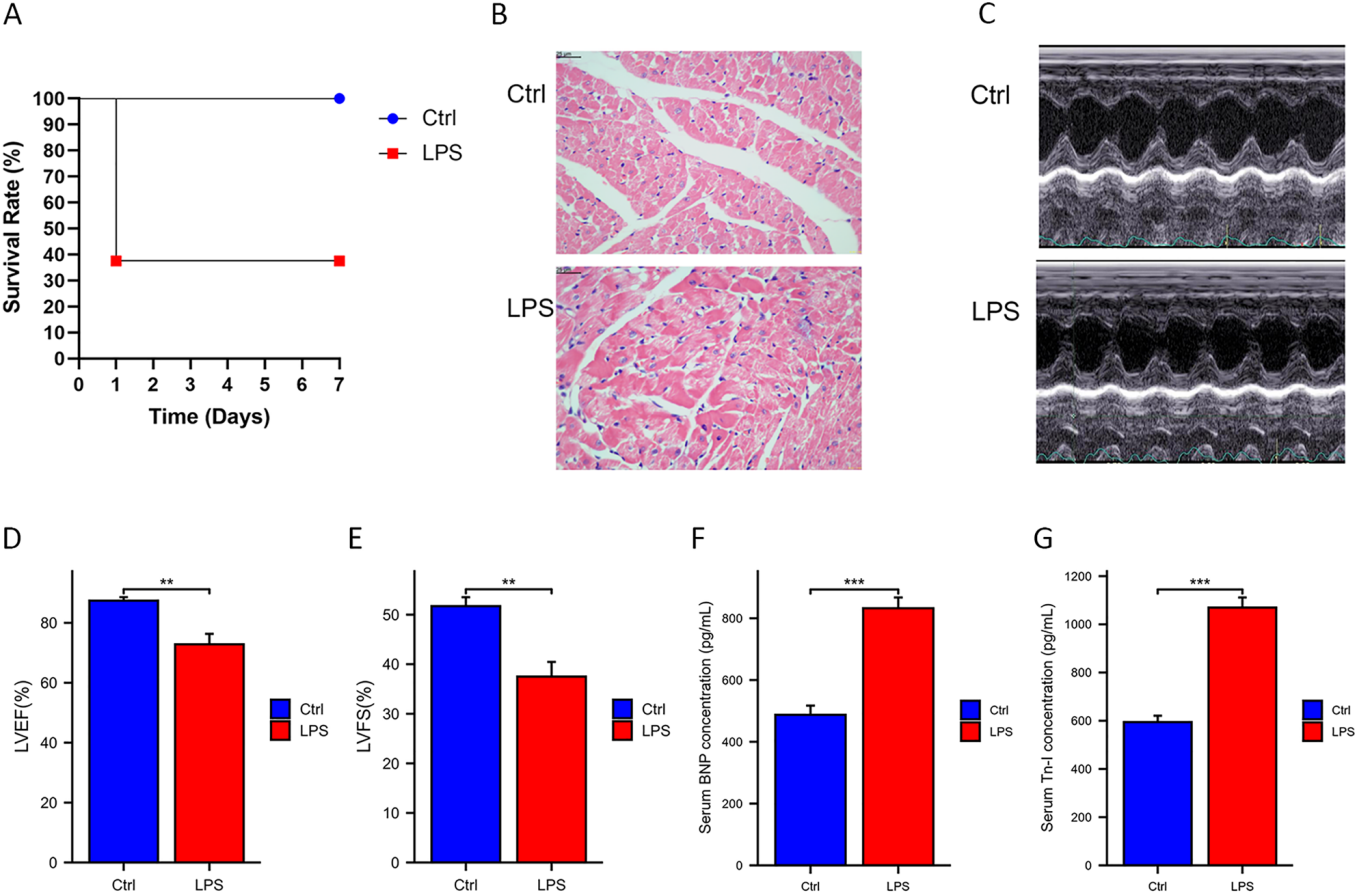
Effect of LPS injection on cardiac function in rats. (**A**) Survival rate of rats in LPS and Ctrl groups (n=5). (**B**) Histological changes of the myocardial tissue assessed by HE staining. Magnification, X400. (**C**) Representative echocardiographic images are shown. Echocardiographic parameters were shown in Supplemental Table 2. (**D**) LVEF. (**E**) LVFS. (**F**) Tn-I. (**G**) BNP. ∗ *P* < 0.05; ∗∗ *P* < 0.01; ∗∗∗ *P* < 0.001. The data were shown as the means ± SD (n = 5).

The mental status of rats in the LPS group was observed. The results showed that the respiratory rate of rats in the LPS group was significantly increased, symptoms such as apathy and drowsiness appeared, and their response to external stimuli was weakened. The survival rate in the LPS group was 37.5% (Fig. 2A) (P = 0.009).

### LPS caused changes in the heart muscle and cardiac function

Morphological changes of myocardial cells in heart sections with HE staining after LPS injection showed that the myocardial cells had uneven borders, random and loose arrangements of myocardial fibers, and myocardial abnormalities. Additionally, inflammatory cell infiltration was present; compared to the Ctrl group, the LPS group showed swelling and degeneration of cardiomyocytes and increased erythrocyte exudation (Fig. 2B). The cardiac function of the rats was measured using echocardiography. At 24 h after LPS injection, the myocardial volume at end-diastole and end-systole was increased in the LPS group (Fig. 2C), whereas LVEF and LVFS were reduced (Fig. 2D,E). The levels of cTnI and BNP were measured to investigate myocardial injury in rats with sepsis. As shown in Fig. 2F and G, the cTnI and BNP levels in the LPS group were higher than those in the Ctrl group.

### Identification of DEGs

**Figure 3:**
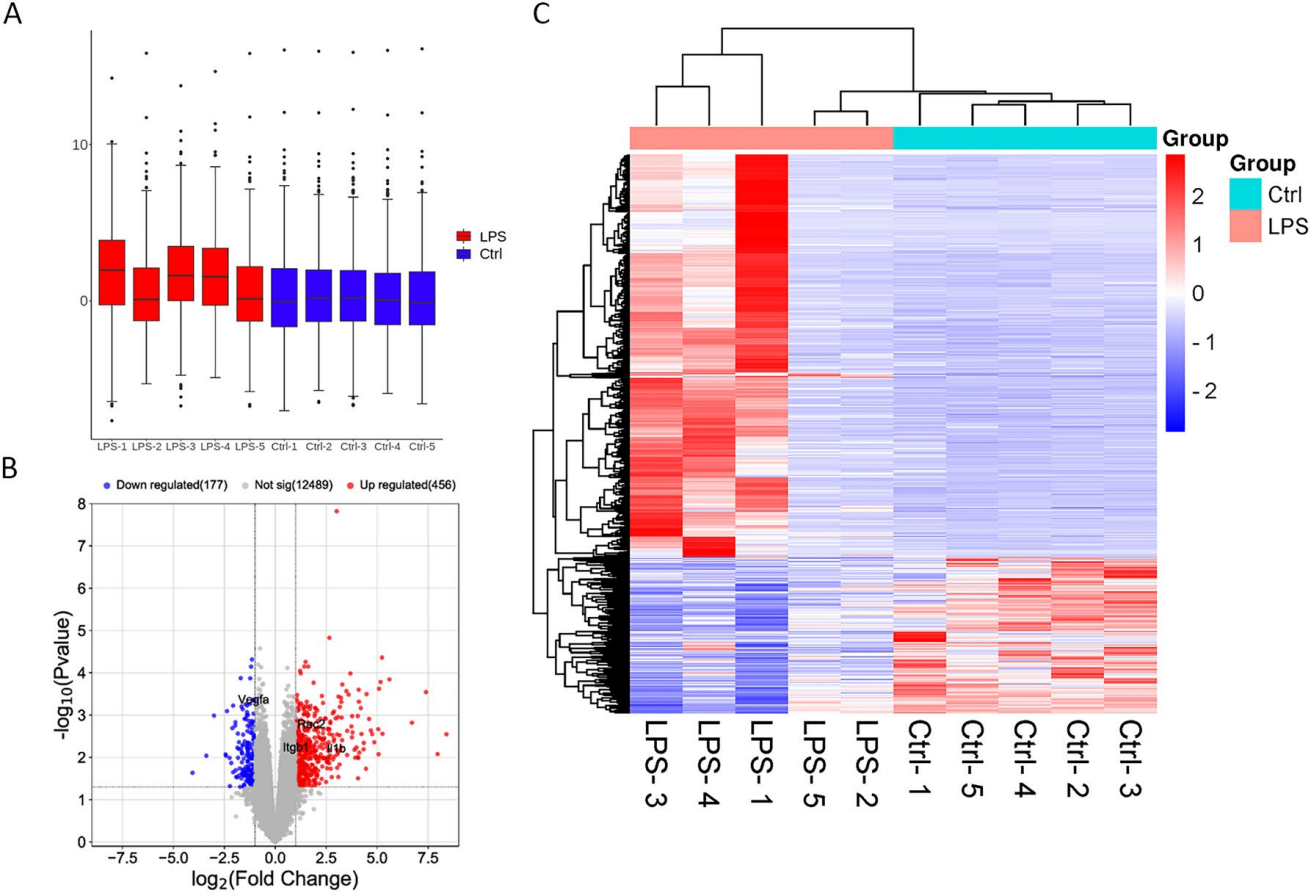
DEGs identification results in RNA-seq. (**A**) Box line graph distribution. The horizontal line in the box was the median of the statistics. (**B**) Volcano map showing the genes expressed in the LPS group detected with RNA-seq. Colored dots indicated genes that do not differ significantly; red dots indicated genes with a log2 FC > 1 and *P* < 0.05; blue dots indicated genes with log2 FC < − 1 and *P* < 0.05. (**C**) Heat map of clustering of DEGs. Red indicated highly expressed genes, and blue indicated lowly expressed genes. The x-axis indicated different samples, and the y-axis indicated gene names.

The box plot of the RNA-seq dataset showed a linear distribution trend (Fig. 3A), indicating data reproducibility. A total of 633 DEGs (Fig. 3B and Supplemental Table 1), of which 456 were up-regulated and 177 were down-regulated, were screened using the criterion of |LPS vs. Ctrl log2^fold change^|≥ 1 and* P* value < 0.05.

An overall hierarchical clustering plot was constructed for the five samples from the LPS group and five samples from the Ctrl group according to the RPKM values of the DEGs. In the clustering heat map (Fig. 3C), the DEGs were clearly distinguished between the Ctrl and LPS groups.

### PPI network analysis and hub genes confirmation

**Figure 4:**
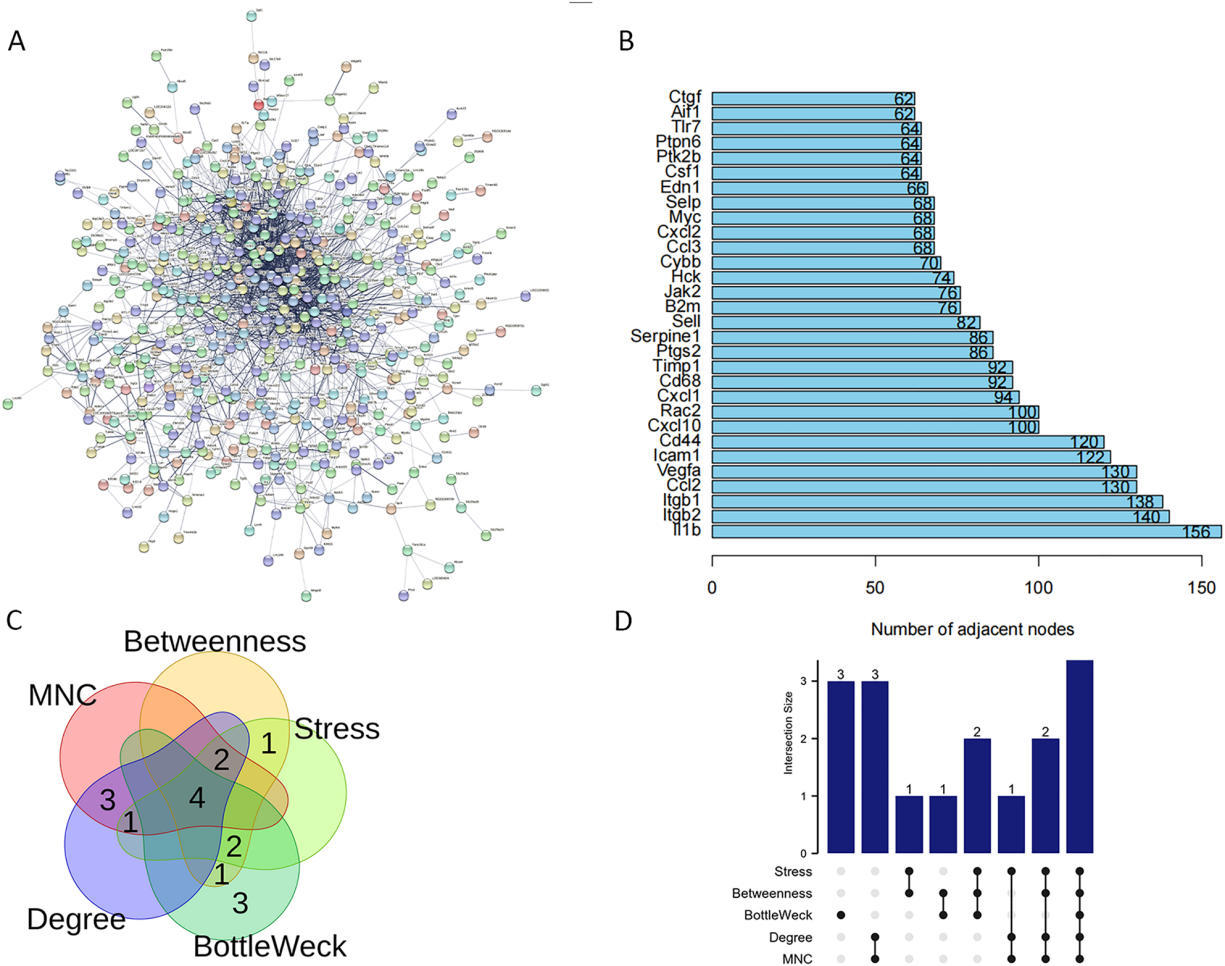
PPI network construction and hub genes analysis. (**A**) PPI network provided by STRING. (**B**) Histogram based on the number of PPI nodes shown in the network (only the first 30 shown). (**C**) Venn diagram showing overlap of DEGs in Ctrl vs. LPS groups. (**D**) The upSet plot of results of CytoHubba.

A PPI network for the DEGs of the RNA-seq dataset was constructed (Fig. 4A) based on the STRING website, showing only the histograms of the top 30 protein–protein interaction nodes (Fig. 4B). The top 10 hub genes of the DEGs were filtered based on five algorithms (MNC, degree, closeness, betweenness, and clustering) using CytoHubba (version 3.10.0) in Cytoscape (Fig. 4C and Supplementary Fig. 1). The UpSetR package (4.2.1) was used to visualize the UpSet plot (Fig. 4D and Supplementary Table 3). Four identified hub genes (*Il1b*, *Rac2*, *Vegfa, Itgb1*) were shown in Table 2.

**Table 2:** Results of the hub genes.

Ensembl ID	Gene ID	Log2FC	*P*-value
ENSRNOG00000010966	Itgb1	1.01984053	0.006028596
ENSRNOG00000004649	Il1b	2.996126274	0.006479263
ENSRNOG00000007350	Rac2	1.771430475	0.001720384
ENSRNOG00000019598	Vegfa	− 1.063170497	0.000450538

### Validation of hub genes

**Figure 5:**
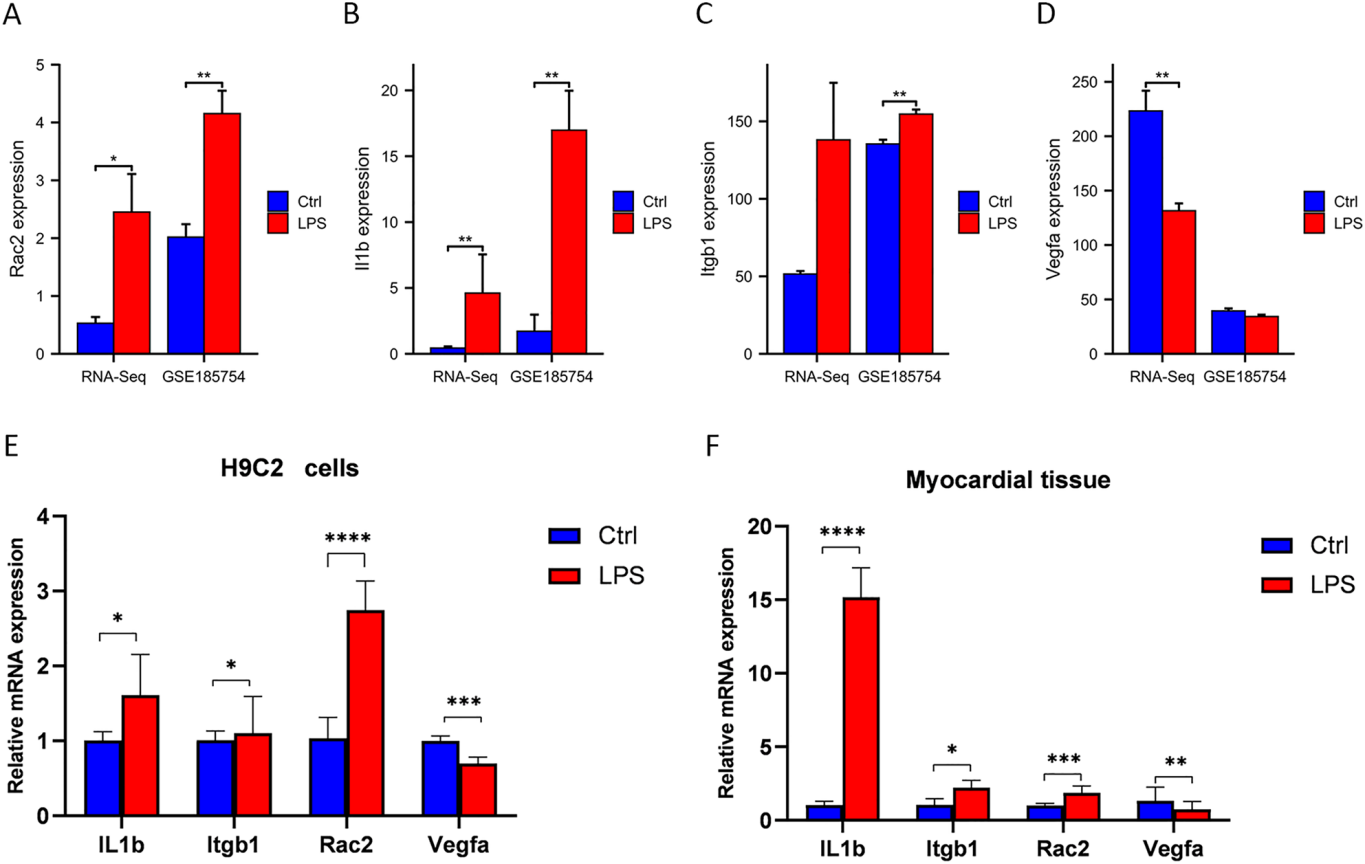
Validation of hub genes in RNA-seq, GSE185754, and RT-qPCR. (**A**–**D**) Comparison of hub genes expression between Ctrl and LPS groups in RNA-seq and GSE185754 datasets. (**A**) Rac2. (**B**) Il1b. (**C**) Itgb1. (**D**) Vegfa. (**E**) RT-qPCR was used to detect the expression of identified hub genes in H9C2 cells (n = 3). Data were the means ± SD of three parallel experiments. (**F**) RT-qPCR was used to detect the expression of identified hub genes in myocardial tissue (n = 5). Data were the means ± SD of five parallel experiments. ∗*P* < 0.05; ∗∗*P* < 0.01; ∗∗∗*P* < 0.001; ∗∗∗*P* < 0.0001.

In the RNA-seq dataset, *Itgb1*, *Il1b,* and *Rac2* were increased, and *Vegfa* was decreased in the LPS group compared to those in the Ctrl group (*P* < 0.05). To verify the accuracy of the RNA-seq results, we selected the GSE185754 dataset and performed RT-qPCR for validation. As shown in Fig. 5A–D, the results of the GSE185754 dataset were consistent with those of our bioinformatics analysis. In myocardial tissue and H9C2 cells, RT-qPCR results were shown in Fig. 5E and F. *Itgb1*, *Il1b,* and *Rac2* were significantly upregulated, and *Vegfa* was significantly downregulated in the LPS group, which was consistent with the RNA-seq and GSE185754 datasets.

### Functional enrichment analysis of hub genes

**Figure 6:**
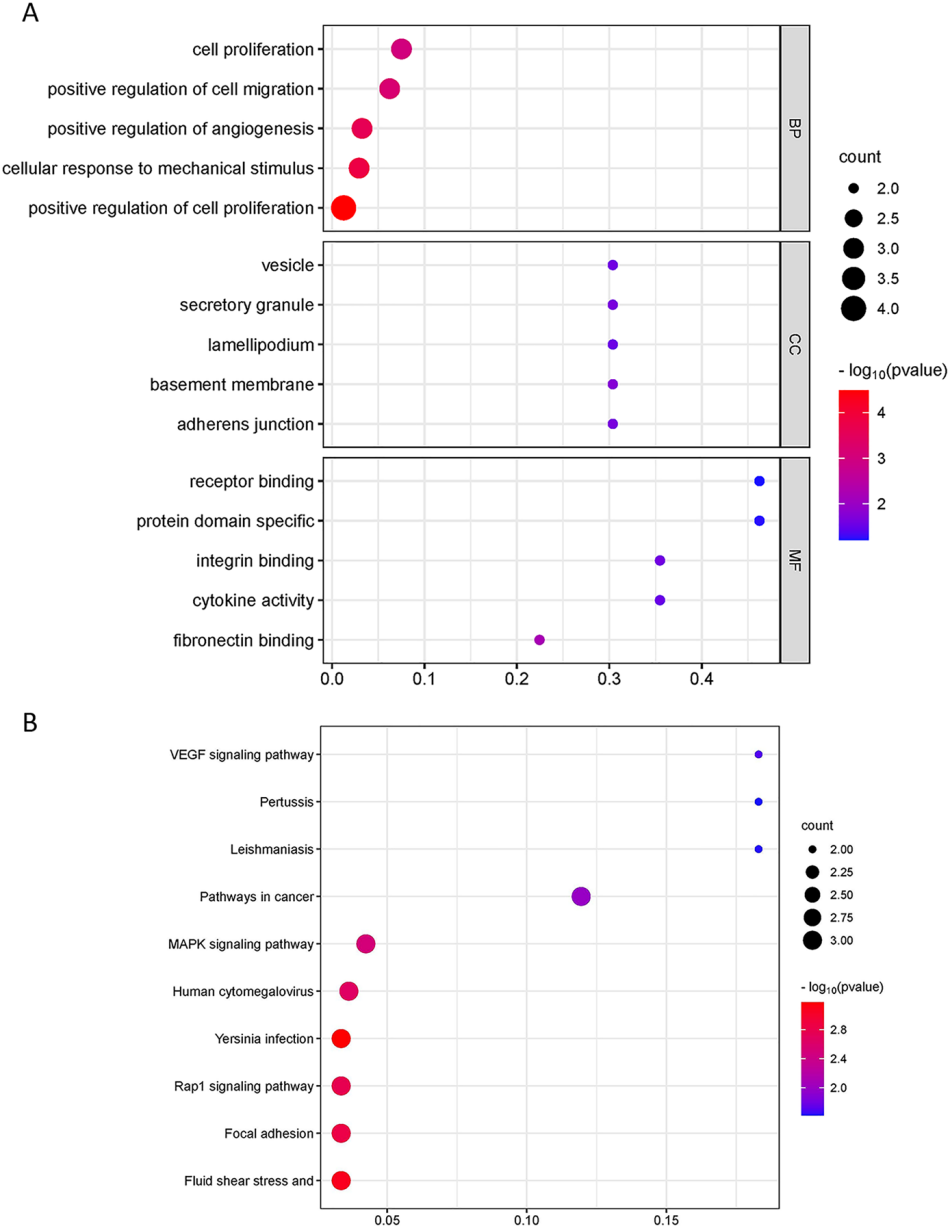
GO/KEGG pathway enrichment of identified hub genes. (**A**) GO categories and pathways of hub genes. (**B**) Top KEGG pathways in the high-expression group of hub genes.

GO/KEGG enrichment analysis and GSEA were performed for four "real" hub genes (*Itgb1*, *Il1b*, *Rac2*, *Vegfa*). As shown in Fig. 6A, in terms of BP, "cell proliferation," "positive regulation of cell migration," "positive regulation of angiogenesis," "cellular response to mechanical stimulus," and "positive regulation of cell proliferation" were enriched. In terms of CC, "vesicle," "secretory granule," "lamellipodium," "basement membrane," and "adherens junction" were enriched. At the same time, the MF terms of hub genes were mainly enriched in "fibronectin binding," "cytokine activity," "integrin binding," "protein domain specificity," and "receptor binding." Furthermore, in the KEGG enrichment analysis shown in Fig. 6B, the hub genes specifically participated in the "Fluid shear stress and atherosclerosis," "Focal adhesion," "Rap1 signaling pathway," "Yersinia infection," and "Human cytomegalovirus infection." According to NES > 1.5 and *P* value < 0.05, samples from RNA-seq were screened for enriched signaling pathways. The RNA-seq samples were subsequently subjected to GSEA based on the log2^fold change^ in hub gene expression. As shown in Table 3, hub genes were enriched in "KEGG_CYTOKINE_CYTOKINE_RECEPTOR_INTERACTION," "KEGG_COMPLEMENT_AND_COAGULATION_CASCADES," "KEGG_CHEMOKINE_SIGNALING_PATHWAY," "KEGG_MAPK_SIGNALING_PATHWAY."

**Table 3:** Results of the GSEA enrichment analysis of the hub genes.

ID	setSize	Enrichment score	NES	*P-*value
KEGG_CYTOKINE_CYTOKINE_RECEPTOR_INTERACTION	24	0.6639429	2.444934	6.34e − 07
KEGG_CHEMOKINE_SIGNALING_PATHWAY	20	0.6166404	2.159886	0.0001
KEGG_COMPLEMENT_AND_COAGULATION_CASCADES	12	0.5755611	1.749990	0.0138
KEGG_MAPK_SIGNALING_PATHWAY	11	0.5677923	1.672900	0.0248

### Evaluation based on identified hub genes

**Figure 7:**
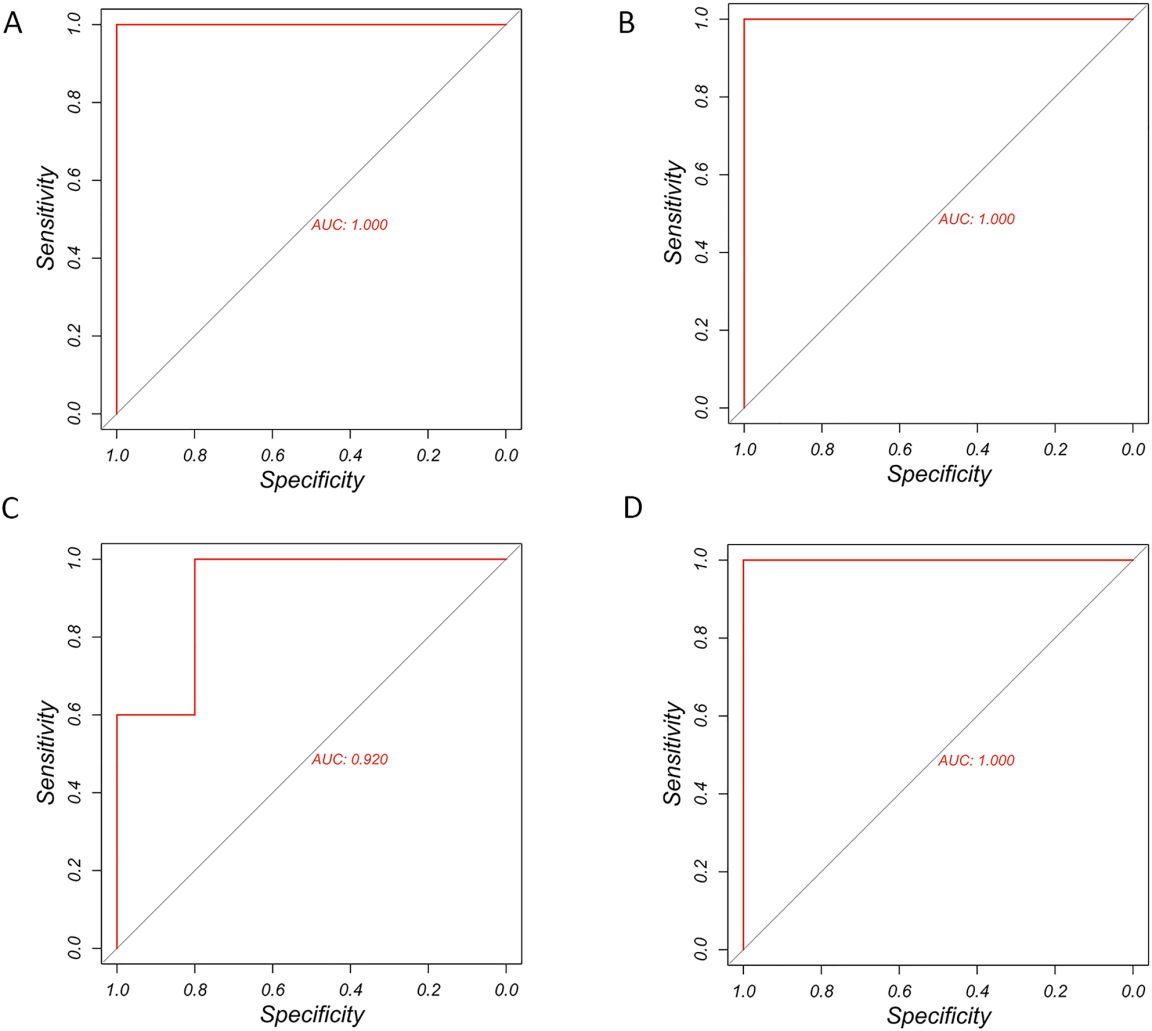
The ROC results for the hub genes. (**A**) Il1b. (**B**) Itgb1. (**C**) Rac2. (**D**) Vegfa.

To verify the diagnostic significance of these four hub genes, we performed ROC curve analysis. The ROC values of Vegfa, Il1b, and Itgb1 were all 1; the ROC value of Rac2 was 0.92 (Fig. 7A–D).

### Construction of mRNA-miRNA regulatory network

**Figure 8:**
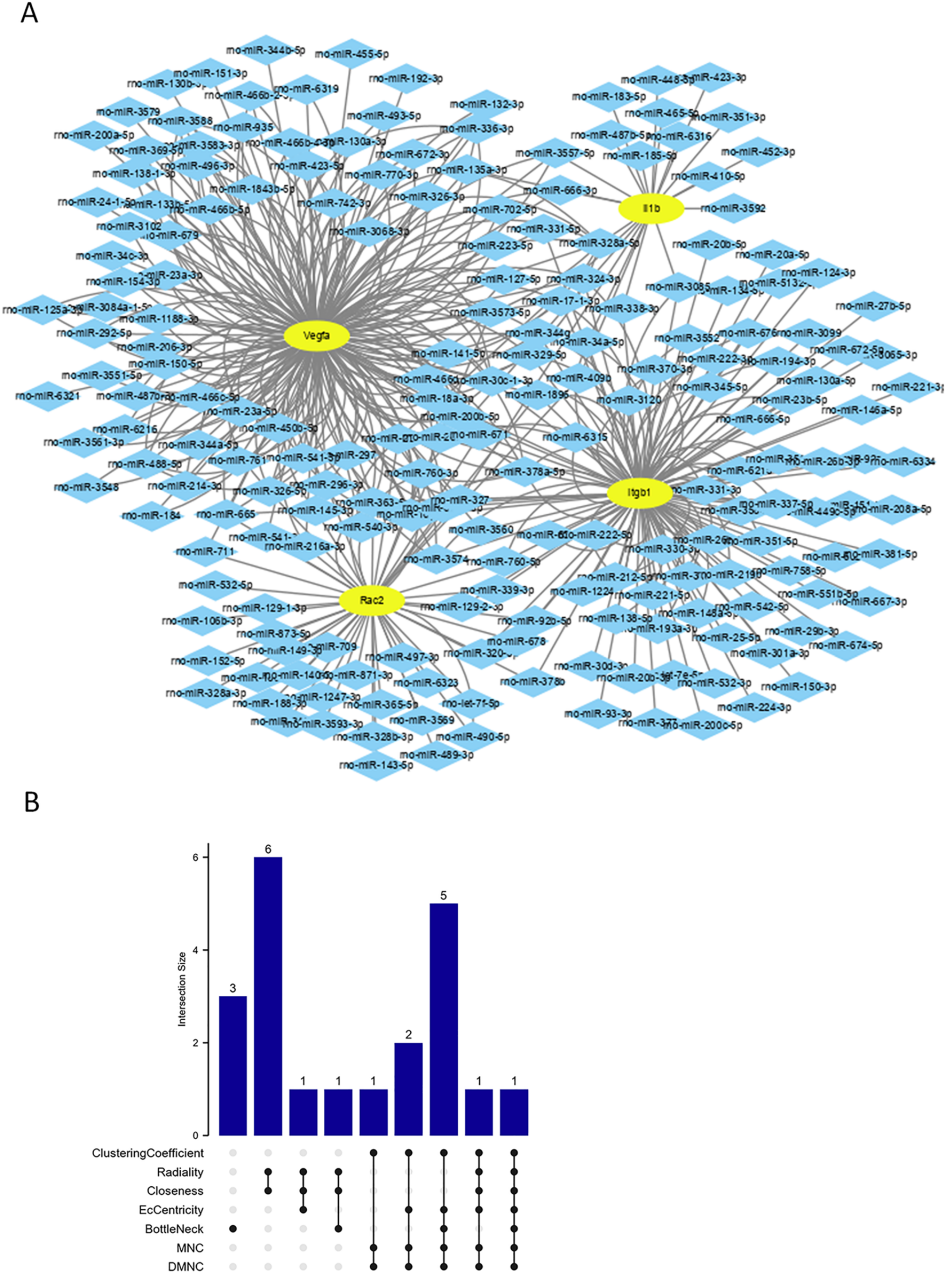
mRNA-miRNA regulatory network of identified hub genes. (**A**) mRNA-miRNA regulatory network presented by STRING. (**B**) The upSet plot of CytoHubba. The top 10 miRNAs for the seven algorithms were shown in Supplementary Figure 2 and Supplementary Table 4.

In total, 461 target miRNAs were predicted at the miRWalk site for the four hub genes. A co-expression network of hub genes and miRNAs was constructed using Cytoscape, with 211 nodes and 461 interactions (Fig. 8A). Finally, six miRNAs were procured at the intersection of the seven algorithms and visualized using the upset package, as shown in Fig. 8B and Table 4.

**Table 4:** miRNA of CytoHubba.

Index	Elements
1	miR-541-5p
2	miR-487b-3p
3	miR-1224
4	miR-378a-5p
5	miR-6334
6	miR-466b-5p

### Targeted drug prediction

**Figure 9:**
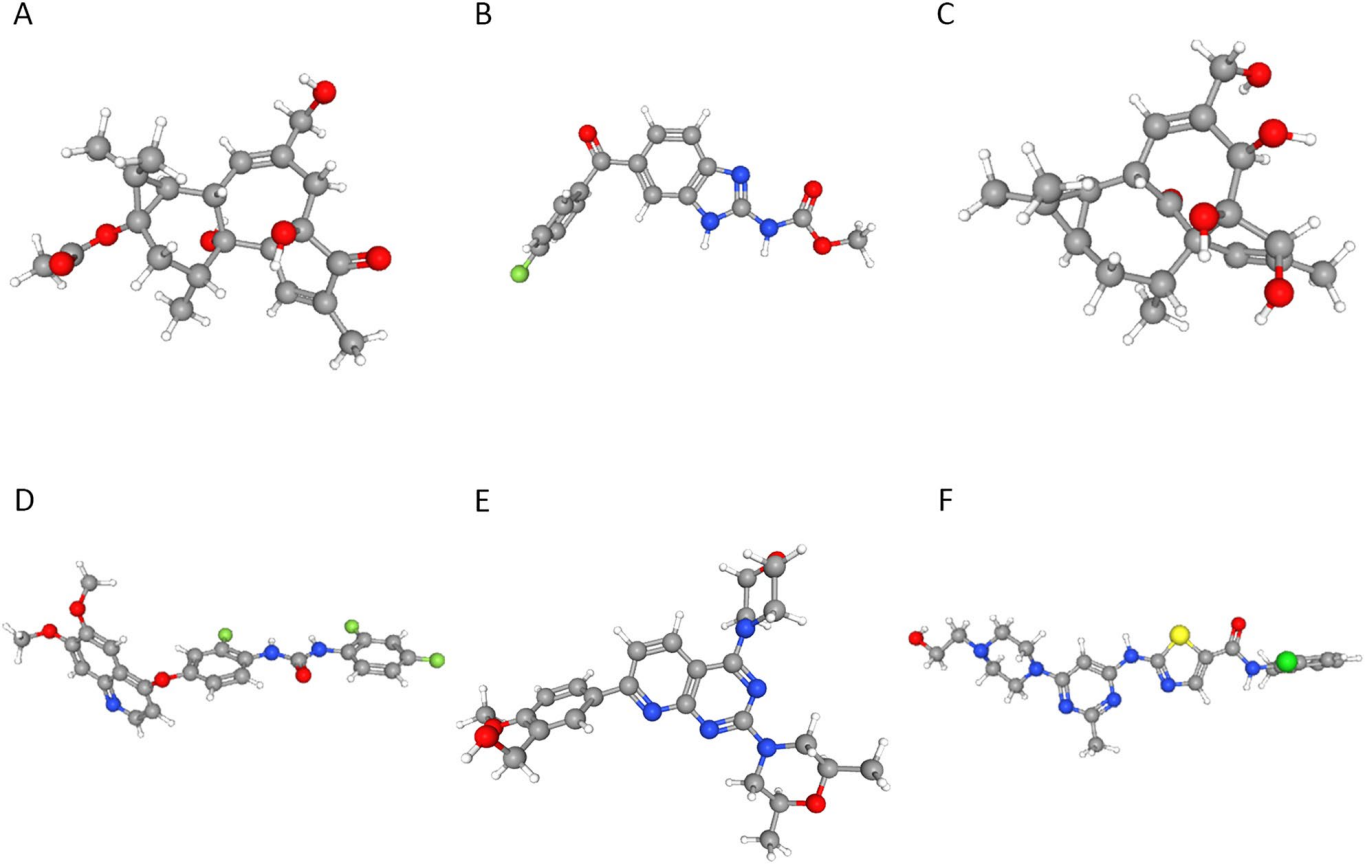
3-D structure of small molecule drugs. (**A**) Prostratin. (**B**) Flubendazole. (**C**) Ingenol. (**D**) KI-8751. (**E**) KU-0063794. (**F**) Dasatinib.

The DEGs were categorized into upregulated or downregulated groups and enriched with significantly altered genes from the CMAP database of small-molecule therapeutics. Target compounds that induced similar or opposite effects in greater than 95% of SCM cases were selected (similar: prostratin, flubendazole, ingenol, KI-8751; opposite: KU-0063794 and dasatinib), as shown in Table 5. Next, the 3-D structures of the small-molecule drugs were obtained using PubChem (Fig. 9A–F).

**Table 5:** Researching results of CMAP.

CMAP name	Score	ID	Target	MOA
Prostratin	98.47	BRD-K91145395	PRKCA, PRKCB, PRKCD, PRKCE, PRKCG, PRKCH, PRKCQ	PKC activator
Flubendazole	95.7	BRD-K86003836	TUBB	Tubulin inhibitor
Ingenol	95.63	BRD-A52650764	PRKCD, PRKCE	PKC activator
KI-8751	95.54	BRD-K47150025	KDR	VEGFR inhibitor, KIT inhibitor, PDGFR receptor inhibitor
KU-0063794	− 95.14	BRD-K67566344	MTOR	MTOR inhibitor
Dasatinib	− 95.57	BRD-K49328571	ABL1, FYN, LCK, SRC, KIT, YES1, BCR, EPHA2, LYN, PDGFRB, ABL2, BTK, DDR1, DDR2, PDGFRA, STAT5B	BCR-ABL kinase inhibitor, Ephrin inhibitor, KIT inhibitor, PDGFR receptor inhibitor, SRC inhibitor, Tyrosine kinase inhibitor

## Discussion

Sepsis was the primary cause of mortality in critically ill patients^12^. SCM was one of the most frequent complications of sepsis with a mortality rate of approximately 80%. Clinical studies had shown that the typical manifestations of SCM were a larger left ventricle, reduced LVEF, cardiac dysfunction, and pathological alteration of cardiac structures^4,13,14^. Varying degrees of myocardial injury can occur in approximately 50% of patients with sepsis. SCM was closely associated with sepsis; once sepsis occurs, it may lead to SCM. Studies had shown that the pathogenesis of SCM involved multiple factors, including the inflammatory response, immune response, abnormal regulation of intracellular calcium transport proteins, and abnormal mitochondrial function^2,15,16^. As the occurrence of SCM was highly reversible, early diagnosis and treatment can significantly improve patient prognosis. The discovery of potential biomarkers was essential for future diagnosis, treatment, and prognosis of SCM.

The gene expression profiles used in this study were taken from the myocardial tissue of a rat model of LPS-induced SCM using RNA-seq technology. *Itgb1*, *Il1b*, *Rac2*, and *Vegfa* were validated as hub genes of SCM using bioinformatics analysis and RT-qPCR. The analysis in this study revealed hub genes and signaling pathways associated with the mechanism of SCM, which were potential biological targets and biomarkers of SCM. Two high-confidence candidate compounds (KU-0063794 and dasatinib) were identified from the CMAP database as new therapeutic drugs for SCM.

A comprehensive analysis based on five algorithms with four hub genes (*Il1b*, *Rac2*, *Vegfa*, *Itgb1*) were identified. *Vegfa* was an important angiogenic factor that selectively enhances mitosis, stimulates endothelial cell proliferation, and promotes angiogenesis^17^. Several studies had shown that LPS-induced macrophage activation upregulated *Vegfa* expression^18,19^. *Vegfa* was upregulated by the LPS-induced expression and secretion of the inflammatory cytokines IL-1β, IL-6, and TNF-α. Lin et al. used cecal ligation puncture (CLP, 24 h) to construct a mouse model of sepsis. They found that low expression of *Vegfa* inhibited LPS-induced M1 polarization in macrophages and demonstrated that reduced *Vegfa* inhibited glycolysis, thereby inhibiting LPS-induced M1 polarization in macrophages and ultimately alleviating sepsis^20^. These findings also supported the notion that *Vegfa* was a potential diagnostic biomarker of SCM. *Rac2* was an essentially small GTP enzyme. *Rac2* was a critical molecule involved in numerous distinct cellular processes, ranging from the recombination of the actin cytoskeleton and lamellipodia formation to gene transcription, cell cycle, and cell growth^21^. Kumar et al. found that increased *Rac2* expression was associated with the pathological processes of organ damage during sepsis^22^. In mice, *Rac2* deficiency had been shown to impair humoral immune responses^21^. These studies were consistent with our study, which showed that *Rac2* can participate in processes such as cardiomyocyte apoptosis, inflammatory response, and myocardial injury, thus affecting the occurrence and development of SCM, which was closely related to the occurrence of SCM. *IL-1β* was a multipotent cytokine, a critical factor in initiating immune responses, and a hub mediator of the inflammatory tumor microenvironment. Busch et al.^23^ induced male mice to SCM caused by polymicrobial sepsis through CLP and found that *IL-1β* caused myocardial atrophy, impaired its contractility and relaxation, and reduced the deformation of cardiomyocytes. Because inhibition of the NLRP3/IL-1*β* pathway slowed myocardial atrophy and cardiomyopathy in sepsis, it can prevent SCM. Our study was consistent with these findings, suggesting that *IL-1β* may be a promising prognostic marker of SCM. In previous clinical studies, *IL-1β* had been identified as a suppressor of the myocardium^24^, and increased serum levels of *IL-1β* were found in SCM rat models and patients with SCM^25,26^. The protein Itgb1 encoded by gene *Itgb1* was a cell surface receptor that acted as a bridge in the basement membrane, extracellular matrix, and cytoskeleton of endothelial cells^27,28^. Zhou et al. used H9C2 cardiomyocytes to show that *Itgb1* regulated the mTORC2/Akt pathway, thereby inhibiting autophagy in cardiomyocytes^29^. Previous studies^30^ had found that LPS-treated H9C2 cells showed reduced autophagic activity, increased mitochondrial membrane potential instability, and apoptosis. However, LPS-induced myocardial injury was attenuated by increased autophagy. These results suggested that *Itgb1* was a potential novel target molecule for the treatment of heart disease. Screening for the post-transcriptional regulators of *Itgb1, Il1b, Rac2,* and *Vegfa,* which can be associated with SCM, provided novel insights into the pathophysiological mechanisms, diagnosis, and therapy of SCM.

Sepsis can strongly activate both hemostatic and complement systems. As sepsis progresses, the blood and tissue barriers can break down because of the systemic response of the host to bacterial pathogen-associated molecular patterns. This response can be exaggerated, resulting in abnormal barriers in the body^31^. Lupu et al. found that live bacteria and bacterial products can trigger complement and coagulation cascades, suggesting that preventing the formation of complement activation products may be a promising therapeutic approach^32^, particularly in cases of severe sepsis when organ failure occurred. Sepsis was a systemic inflammatory response characterized by a significantly imbalanced cytokine response, called a cytokine storm^16^. Recent studies had shown that targeting cytokine storms can significantly alleviate tissue damage and assist in the clearance of invading pathogens^33^. During severe sepsis, the coagulation system was activated by pro-inflammatory cells, cytokines, and chemokines, which can downregulate critical physiological anticoagulation mechanisms. Consequently, fibrin production increases, catabolism was impaired, and (micro)vascular clot deposition may occur, leading to tissue ischemia, organ dysfunction, and complications^34^. The MAPK signaling pathway was a significant factor in myocardial injury caused by sepsis^34^. MAPKs were serine and threonine kinases that regulated the activity of transcription factors involved in cell differentiation, proliferation, and death. It was crucial to induce the expression of proinflammatory mediators and cytokines^35^. LPS triggered the MAPK signaling pathway, leading to an increase in MAPK expression in lymphocytes. LPS also enhanced Th1 cell-mediated cellular immunity^36^. Studies had shown that the inhibition of the MAPK signaling pathway can provide a protective effect in septic organs^37–39^. The results of the signaling pathways enriched by GSEA were consistent with these findings.

MiRNAs were recognized as important factors in the development of various diseases. Aberrant miRNA expression had been associated with a variety of pathological conditions including cardiovascular disease^40^. The study had found that miR-541-5p was a key effector in acute lung injury tissue caused by the LPS^41^. It was shown that miR-1224 increased the activity of NF-κB in cells, thus providing a new perspective on the regulatory mechanism of the innate immune response^42^. MiR-487b-3p was a member of the miR-487b family, and miR-487b can be used as a potential diagnostic marker for cardiovascular diseases^43,44^. Furthermore, miR-378a-5p, miR-6334, and miR-466b-5p were miRNAs, and miRNAs can regulate the translation and stability of the targeted mRNA^45^.

Additionally, CMAP filtered potential drugs that induced or reversed DEGs expression. The potential therapeutic agents included KU-0063794 and dasatinib. KU-0063794 was a mTORC1/2 inhibitor. Abe et al.^46^ induced necrosis in H9C2 cells by TNF-α and z-VAD-fmk, proving that mTORC1 inhibition promoted autophagy and protected cardiomyocytes from necroptosis. Transcriptome studies had revealed significant changes in glycolysis and mTORC/HIF-1α signaling pathways during sepsis^47^. Dasatinib was a multi-kinase inhibitor with substantial effects on Src family tyrosine kinases, acting on ASrc/Abl tyrosine kinases^48^. In addition to its effect on malignant cells, dasatinib reduced systemic TNF-α production after LPS injection in an Src and Bruton tyrosine kinase-dependent manner^49^. According to the study conducted by Gonçalves-de-Albuquerque et al.^50^, dasatinib enhanced survival and sepsis severity in mice in various microbial sepsis models.

However, bioinformatics analysis had some limitations. First, there were few databases on rat models of SCM induced by LPS injection at 24 h; hence, the GSE185754 dataset on mice was used to validate the results. Second, the current study was restricted by inadequate experimental verification of identified hub genes; the mechanism of the SCM hub genes must be validated in subsequent experiments. Finally, these recognized biomarkers were restricted to a theoretical degree; additional experimental studies and clinical trials should be conducted to obtain accurate validation and validate our results to provide theoretical support for clinical treatment.

We had successfully identified four "real" hub genes (*Itgb1, Il1b, Rac2, Vegfa*) and six key miRNAs, associated with SCM. Anomalies in cytokine-cytokine receptor interactions, complement and coagulation cascades, chemokine signaling pathways, and MAPK signaling pathways had been suggested to play vital roles in the pathogenesis of SCM. KU-0063794 and dasatinib offered novel insights into the treatment of patients with SCM. Further validation experiments on gene functions should be designed to test the potential mechanisms of action of these hub genes and their associated pathways.

### Supplementary Information

Supplementary Figure 1.

Supplementary Figure 2.

Supplementary Table 1.

Supplementary Table 2.

Supplementary Table 3.

Supplementary Table 4.

## Data Availability

The corresponding authors can grant datasets formed and sifted in this study upon sanity demand.
