# Supplementary material for: Exploring potential therapeutic agents for lipopolysaccharide-induced septic cardiomyopathy based on transcriptomics using bioinformatics
Source: Sci Rep. 2023 Nov 23;13:20589. doi: 10.1038/s41598-023-47699-0 (PMC10667505; doi:10.1038/s41598-023-47699-0)
Supplement: Supplementary file 1 — Supplementary Figure 1. [file 41598_2023_47699_MOESM1_ESM.docx]

A Stress

B Betweenness


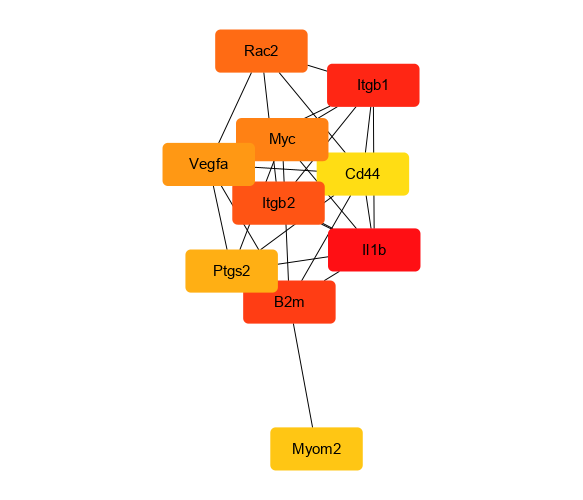


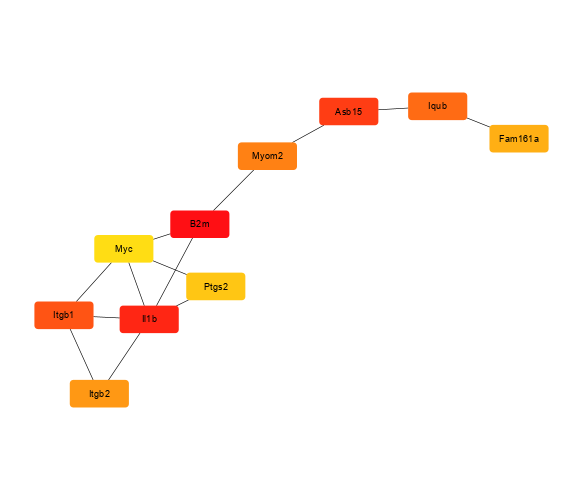


D Degree

C BottleWeck


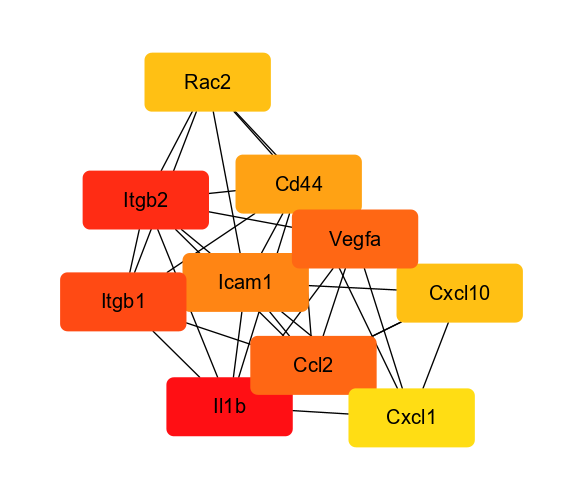

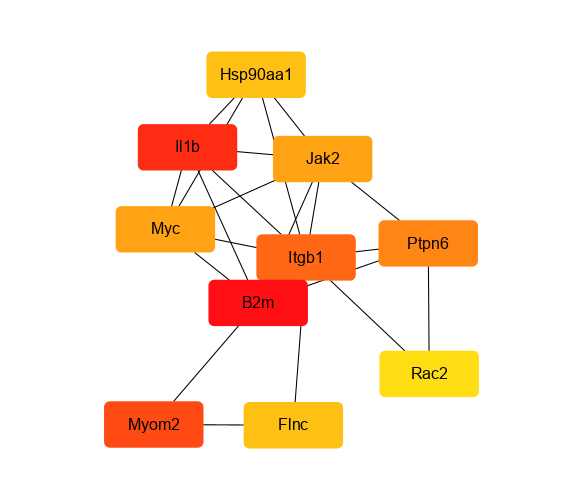


E MNC


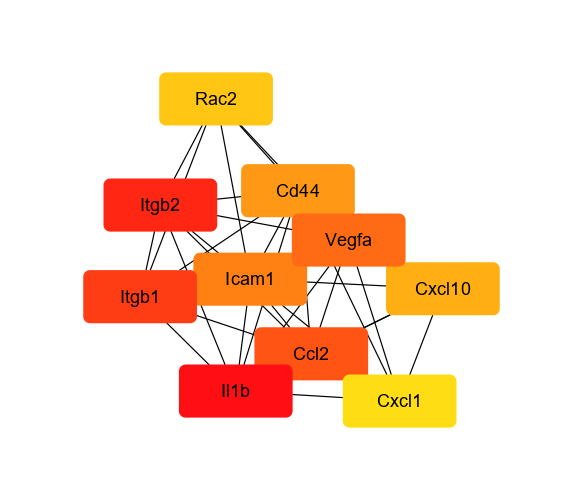


**Supplementary Figure 1:** hub genes shown by CytoHubba from five algorithms.
