# Supplementary material for: Exploring potential therapeutic agents for lipopolysaccharide-induced septic cardiomyopathy based on transcriptomics using bioinformatics
Source: Sci Rep. 2023 Nov 23;13:20589. doi: 10.1038/s41598-023-47699-0 (PMC10667505; doi:10.1038/s41598-023-47699-0)
Supplement: Supplementary file 2 — Supplementary Figure 2. [file 41598_2023_47699_MOESM2_ESM.docx]

B Radiality

A ClusteringCoefficient


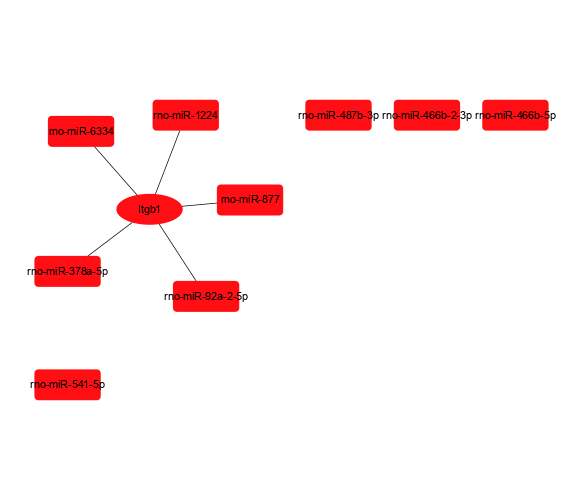

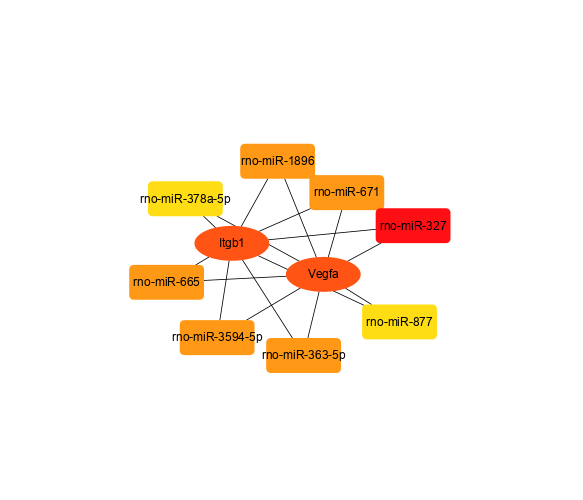


C Closeness

D EcCentricity


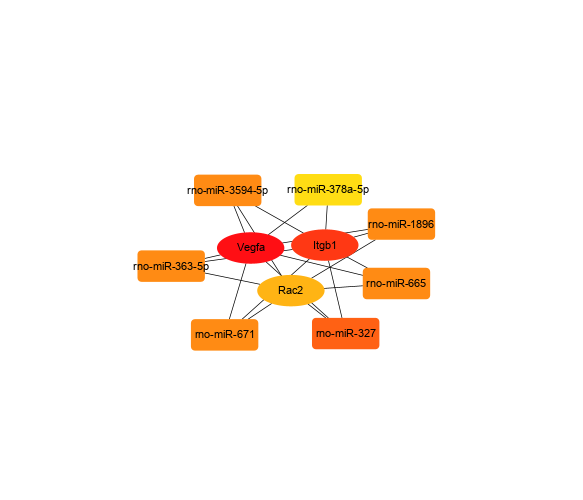

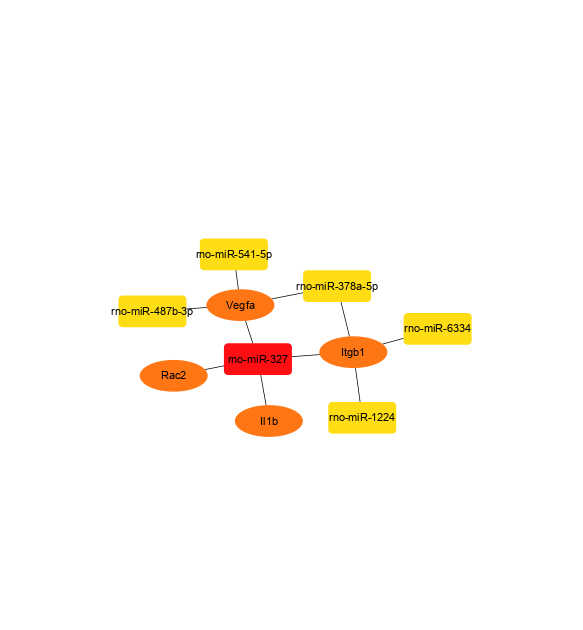


F DMNC

E BottleNeck


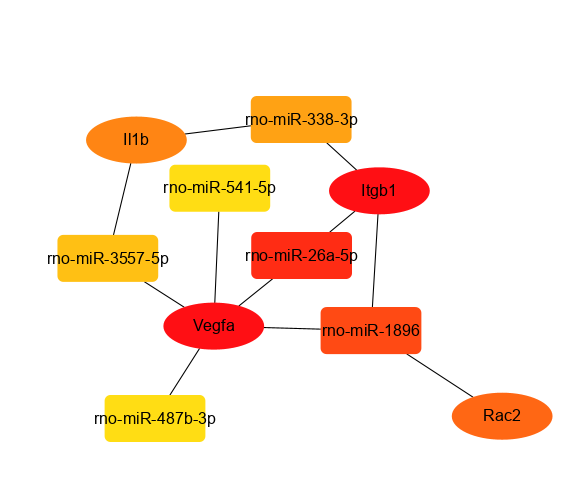

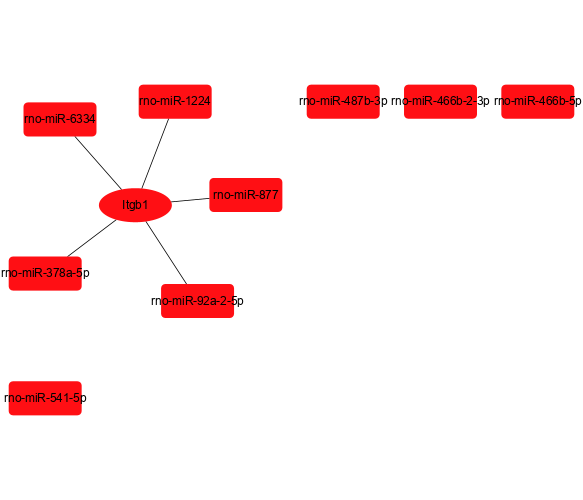


G MNC


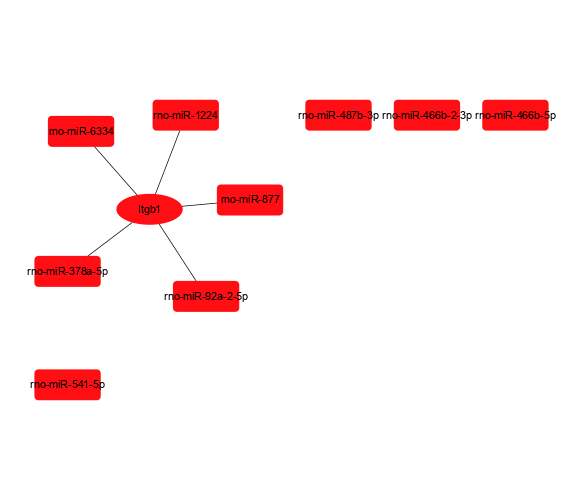


**Supplementary Figure 2:** Hub miRNA targeted identified hub genes shown by CytoHubba.
